# Adjuvant immunotherapy improves survival in completely resected stage IB–III NSCLC: a systematic review and meta-analysis

**DOI:** 10.3389/fonc.2025.1493221

**Published:** 2025-04-08

**Authors:** Hong Huang, Pengchen Bao, Hongyu Jin, Wenyang Li, Hui Shen, Zhen Qin, Ying Pan, Xinming Su, Delei Kong

**Affiliations:** ^1^ Department of Pulmonary and Critical Care Medicine, The First Hospital of China Medical University, Shenyang, China; ^2^ China Medical University, Shenyang, China; ^3^ Department of Laboratory Medicine, The First Hospital of China Medical University, Shenyang, China

**Keywords:** adjuvant immunotherapy, immune checkpoint inhibitors, ICIs, non-small cell lung cancer, NSCLC

## Abstract

**Background:**

The clinical benefits of postoperative chemotherapy for non-small cell lung cancer (NSCLC) have plateaued, thus highlighting the need for novel strategies. This meta-analysis evaluated the efficacy and safety of adjuvant immunotherapy in patients with completely resected NSCLC and wild-type epidermal growth factor receptor (EGFR) or anaplastic lymphoma kinase (ALK).

**Methods:**

PubMed, Web of Science, Embase, and the Cochrane Library were searched up to February 12, 2025, for studies assessing adjuvant immunotherapy in NSCLC. Primary endpoints included disease-free survival (DFS), overall survival (OS), correlation between subgroup characteristics and efficacy, and safety outcomes, including treatment-related adverse events (TRAEs), severe adverse events (SAEs), and treatment discontinuation.

**Results:**

Twelve articles involving 4048 patients were included. Adjuvant immunotherapy significantly improved DFS in patients with resected stage IB–III NSCLC than supportive care or placebo (hazard ratio [HR]: 0.82, 95% confidence interval [CI]: 0.72–0.93, *p* = 0.01; I^2^ = 0%, *p* = 0.46). However, the OS benefit was not significant (HR: 0.9, 95% CI: 0.67–1.21, *p* = 0.34). DFS benefit was observed in EGFR-negative (HR: 0.75, 95% CI: 0.62–0.91, I^2^ = 0%), EGFR status unknown (HR: 0.78, 95% CI: 0.63–0.96, I^2^ = 0%), programmed cell death ligand 1 (PD-L1) 1–49% (HR: 0.75, 95% CI: 0.58–0.97, I^2^ = 7.13%), non-squamous cell carcinoma (HR: 0.72, 95% CI: 0.61–0.84, I^2^ = 0%), and never-smoking (HR: 0.68, 95% CI: 0.49–0.96, I^2^ = 0%) subgroups. The pooled incidences of TRAEs, SAEs, and discontinuation of treatment due to toxicity were 70% (95% CI: 62%–77%), 12% (95% CI: 8%–16%), and 17% (95% CI: 15–19%), respectively.

**Conclusions:**

Adjuvant immunotherapy improved DFS in patients with completely resected NSCLC, particularly those who were EGFR-negative, had PD-L1 levels of 1–49%, had non-squamous cell carcinoma, or never smoked.

**Systematic Review Registration:**

https://www.crd.york.ac.uk/PROSPERO, identifier CRD42024547752.

## 1 Introduction

Lung cancer is a leading cause of cancer-related deaths worldwide (1). The 5-year survival rate of patients with non-small cell lung cancer (NSCLC) remains poor (2). Complete surgical excision is the primary approach to achieving long-term survival. However, postoperative recurrence occurs in 28–68.2% of patients with completely resected NSCLC (3–5), and only 37.1% of them receive standard adjuvant chemotherapy (6). However, the 5-year overall survival (OS) rate for those receiving postoperative chemotherapy remains between 44.5% and 69% (7–10). Therefore, novel individualized adjuvant treatments are needed to decrease postoperative recurrence and improve survival in patients with radically resected NSCLC.

Wu et al. (11) found that compared to placebo, osimertinib significantly improved the disease-free survival (DFS) of patients with epidermal growth factor receptor (EGFR) mutation-positive, completely resected NSCLC (hazard ratio [HR]: 0.20, *p* < 0.001). In addition, the ALINA trial results demonstrated that alectinib therapy significantly improved DFS in patients with completely resected NSCLC harboring anaplastic lymphoma kinase (ALK) rearrangement compared to standard adjuvant chemotherapy (12). Therefore, the National Comprehensive Cancer Network guidelines recommend osimertinib and alectinib use in patients with EGFR and ALK mutations, respectively. Real-world data suggest that the efficacy of postoperative EGFR-tyrosine kinase inhibitors (TKIs) alone is equivalent to that of adjuvant chemotherapy followed by EGFR-TKIs (13).

However, the efficacy of immune checkpoint inhibitors (ICIs) administered to patients with NSCLC after surgical resection is inconsistent. Felip et al. (14) reported that atezolizumab could significantly prolong DFS in patients with resected stage II–IIIA NSCLC after standard adjuvant chemotherapy, with the most significant benefit observed in those with programmed cell death ligand 1 (PD-L1) ≥ 50%. Conversely, a real-world study by Yang et al. (15, 16) indicated that adjuvant immunotherapy was not statistically significant. Besse et al. (17) showed that adjuvant pembrolizumab significantly improved DFS in patients with stage II–IIIA NSCLC but not in those with PD-L1 ≥ 50%. Another study reported that 50% of patients undergoing adjuvant immunotherapy experienced grade 3 or higher adverse events (AEs) (18).

Given these conflicting findings, it remains unclear whether the variability in adjuvant immunotherapy efficacy is associated with PD-L1 positivity and expression levels. Furthermore, histological subtypes may influence treatment response. Smoking is a well-established risk factor for lung cancer; however, its impact on adjuvant immunotherapy efficacy remains unexplored. To address these uncertainties, we conducted a meta-analysis summarizing the available evidence up to February 12, 2025.

## 2 Materials and methods

### 2.1 Literature search

This study was conducted in accordance with the preferred reporting items for systematic reviews and meta-analyses (PRISMA) reporting checklist (19). The study was registered in the International Prospective Register of Systematic Reviews (PROSPERO) (CRD42024547752) before data extraction. Two investigators (Hong Huang and Hongyu Jin) independently searched PubMed, Web of Science, the Cochrane Library, and Embase until February 12, 2025. The search terms included (“surgery” or “lobectomy” or “resection”), (“adjuvant immunotherapy” or “adjuvant immune checkpoint inhibitor”) and (“non-small cell lung cancer” or “NSCLC” or “carcinoma, non-small cell lung”). The reference lists of the retrieved publications were reviewed to identify additional eligible articles. Recent abstracts and data from international conferences, including the American Association for Cancer Research, the American Society of Clinical Oncology, and the European Society for Medical Oncology, were reviewed. The search was limited to publications in English.

### 2.2 Selection criteria

Articles meeting the following criteria were included: (i) studies on patients with completely resected stage I–III NSCLC; (ii) use of immune checkpoint inhibitors used after surgery; and (iii) reported outcomes such as DFS, OS, incidence of treatment-related adverse events (TRAEs), severe adverse events (SAEs), and discontinuation of adjuvant immunotherapy due to TRAEs. If multiple articles were derived from the same trial, the one reporting the most recent data or the largest sample size was selected for inclusion. Meta-analyses, reviews, and case reports were excluded. Two reviewers independently screened and assessed the studies, and any disagreements were resolved through discussion.

### 2.3 Outcome measures and data collection

The primary outcomes were DFS and OS. Secondary outcomes included the incidence of TRAEs, SAEs, and treatment discontinuation due to toxicity. Hong Huang and Pengchen Bao independently reviewed all articles. Data on population characteristics, study design, adjuvant treatment regimens, endpoints, and other study details were extracted using templated forms. Delei Kong was consulted to resolve disagreements. We contacted the corresponding authors for missing data when needed.

### 2.4 Quality assessment

Huang and Jin independently performed a quality assessment. Discrepancies were discussed with Delei Kong, who made the final decision. Non-randomized studies were assessed using the ROBINS-I tool (20). The Cochrane risk-of-bias tool was applied to randomized controlled trials (RCTs).

### 2.5 Statistical analysis

Stata version 16.0 was used to analyze the data. The risk of bias figure was created using RevMan 5.3.5. The pooled HR for DFS and OS were calculated. Non-comparative binary data of AEs were pooled as N (%) with a 95% confidence interval (CI). Random-effects models (Restricted Maximum Likelihood) and Knap–Hartung adjustments were used (21). The chi-square test (χ^2^) and inconsistency test (I^2^) were applied to evaluate the statistical heterogeneity between the articles. *P* < 0.1, or I^2^ > 50% indicated significant heterogeneity. A sensitivity analysis was performed by omitting one study at a time to explore the sources of heterogeneity and assess the reliability of the results. A fixed-effects model was used for additional sensitivity analysis when there was no heterogeneity (I^2^ = 0%). Publication bias was assessed using forest plots and Egger’s tests. The explanation for this inconsistency follows the prescription in the Cochrane Handbook. Statistical significance was set at P < 0.05. significant.

## 3 Results

### 3.1 Literature selection

**Supplementary Figure S1** presents the details of the literature screening process. The search yielded 2187 articles. After removing 667 duplicates, 1459 studies were excluded based on titles and abstracts. Sixty-one full-text studies and conference abstracts were carefully reviewed for eligibility. Eleven articles were excluded as they did not include immunotherapy as a postoperative adjuvant regimen. Twenty-nine studies were excluded because they did not report the relevant outcomes. Three conference papers and one article were excluded because their data had already been published in Felip 2021 (15), O’Brien 2022 (22), and Shin 2024 (23). Five studies (18, 24–27) were excluded because they included patients with incomplete resection.

Finally, 12 articles (14–17, 22, 23, 28–33) from 8 clinical trials, covering 4048 patients, were included in the meta-analysis. The characteristics of the included clinical trials are summarized in **Table 1**. Patients with EGFR-positive tumors were excluded from studies by Provencio et al. (29, 30) (28), Chaft et al. (31), and Carbone et al. (32). However, in the Felip 2021 (15) and O’Brien 2022 (22) studies, 11.6% and 6.2%, of patients were EGFR-positive, respectively. In these two studies, 52.4% and 36.9 of the patients were EGFR mutation-negative, whereas the mutation status of the remaining patients was unknown. The immunotherapy regimens included atezolizumab, pembrolizumab, nivolumab, camrelizumab, durvalumab, sintilimab, tislelizumab, and toripalimab. A summary of the risk of bias in RCTs and non-RCTs is provided in **Supplementary Figure S2** and **Supplementary Table S2**.

**Table 1:** Study characteristics of clinical trials.

Study	Clinical trial	Study phase	Study design	Sample size	Period	Main inclusion criteria	Experimental treatment	Control treatment	Primary endpoint
Felip 2021 (15) Felip 2023 (14)	IMpower010	3	Multicenter, randomized,	Test group:507;Control group:498	Oct 2015–Sept, 2018	IB (tumors ≥4 cm) to IIIA	4 cycles of chemotherapy +16cycles atezolizumab	4 cycles of chemotherapy +supportive care	DFS (stage II–IIIA with PD-L1-positive, stage II–IIIA, and ITT)
O’Brien 2022 (22) Besse 2023 (17)	KEYNOTE-091	3	Randomized	Test group:590;Control group:587	Jan 2016–May 2020	IB (tumors ≥4 cm) to IIIA	18 cycles of pembrolizumab	Placebo	DFS (ITT and PD-L1 ≥ 50%)
Provencio 2023 (28)	NADIM II	2	Randomized	Test group:50; Control group:17	Jun 2019–Feb2021	III	nivolumab 480 mg once q4w for 6 months	three observation visits	pathological complete response
Chaft 2022 (31) Carbone 2023 (32)	LCMC3	2	Single-Arm	137	Apr 2017 to Feb2020	IB–IIIB	permitted to receive standard-of-care adjuvant chemotherapy ± thoracic radiotherapy, then receive atezolizumab for up to 12 months.	permitted to receive standard-of-care adjuvant chemotherapy ± thoracic radiotherapy	major pathological response
Yang 2024 (16)	NeoR-World	–	Multicenter, retrospective cohort study	Test group:231; Control group:138	Between Jan 2010 and Mar2022	I–III	Immunotherapy or immunotherapy+ chemotherapy*	Chemotherapy or none	DFS
Provencio 2020 (30) Provencio 2022 (29)	NADIM	2	Multicenter, Single-Arm	37	Apr 2017–Aug 2018	IIIA	Nivolumab 240 mg q2w for 4 months followed by 480 mg q4w for 8 months	–	PFS at 24 months
Shin 2024 (23)	–	2	Single center Single-Arm	37	Oct 2017–Dec 2023	IIIA–N2	Pembrolizumab 200mg q3w for 2 years	–	DFS
Goss 2024 (33)	BR.31	3	Multicenter, randomized,	1219	May 2015–Aug 2024	IB–IIIA	Durvalumab q4w for 12 months	Placebo	DFS (PD-L1 TC>25% with EGFR^-^/ALK^-^)

AC, adjuvant chemotherapy; DFS, disease-free survival; PFS, progression-free survival; ITT, intention-to-treat. *Immunotherapy regimens in this study included camrelizumab, durvalumab, nivolumab, pembrolizumab, sintilimab, sintilimab + nivolumab, tislelizumab, and toripalimab.

### 3.2 Clinical efficacy of adjuvant immunotherapy

**Figure 1:**
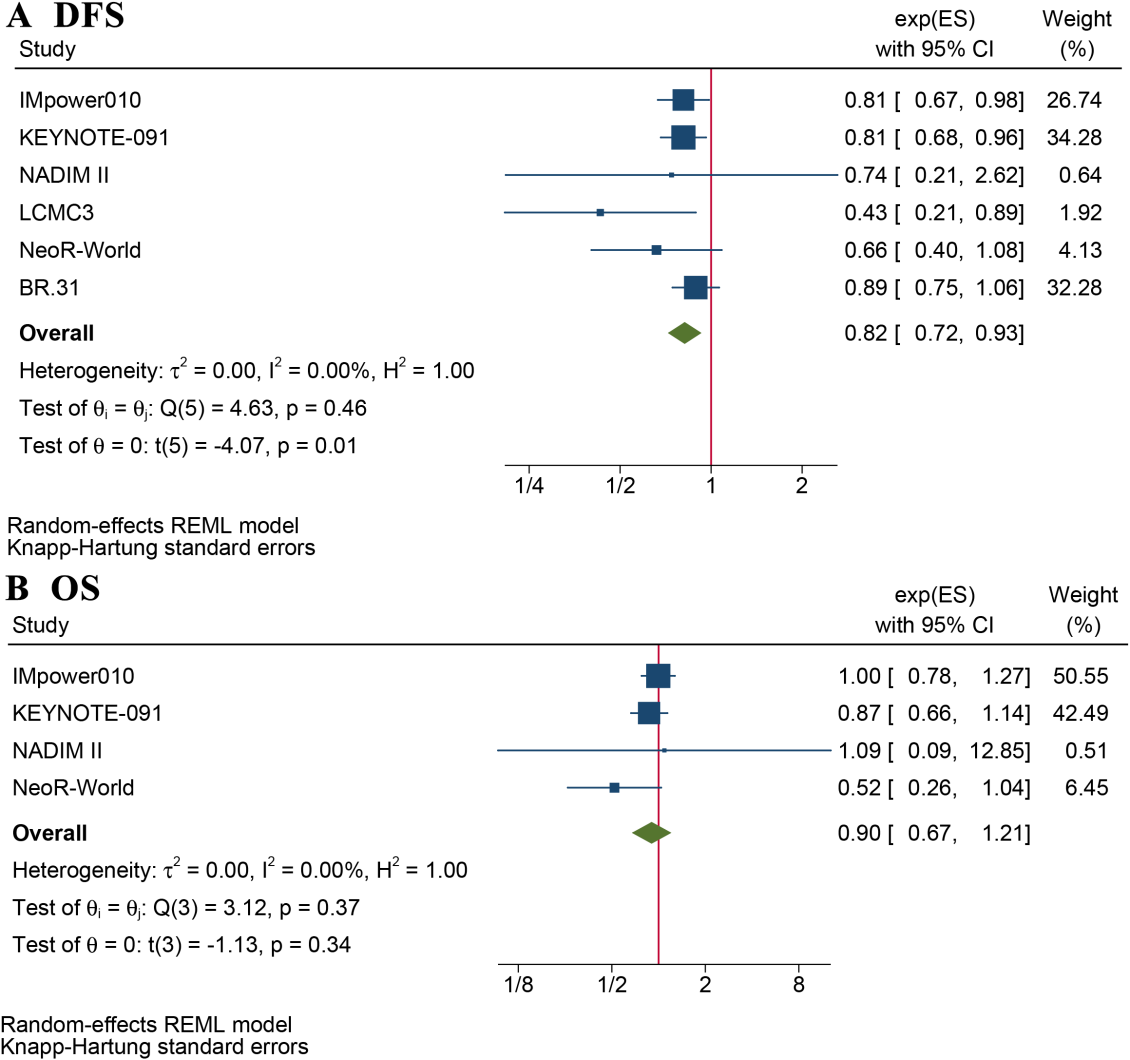
Forest plot of disease-free survival **(A)** and overall survival **(B)**.

Eight articles reported DFS and/or OS (**Supplementary Table S3**). The mean follow-up ranged from 21.6 –71 months. The DFS benefit of adjuvant immunotherapy treatment in completely resected stage IB–III NSCLC was statistically significant (HR: 0.82, 95% CI: 0.72–0.93, *p* = 0.01), without statistical heterogeneity (I^2^ = 0%, *p* = 0.5), as shown in **Figure 1A**. The OS benefit was not statistically significant (HR 0.90, 95% CI: 0.67–1.21, *p* = 0.34), as shown in **Figure 1B**.

**Figure 2:**
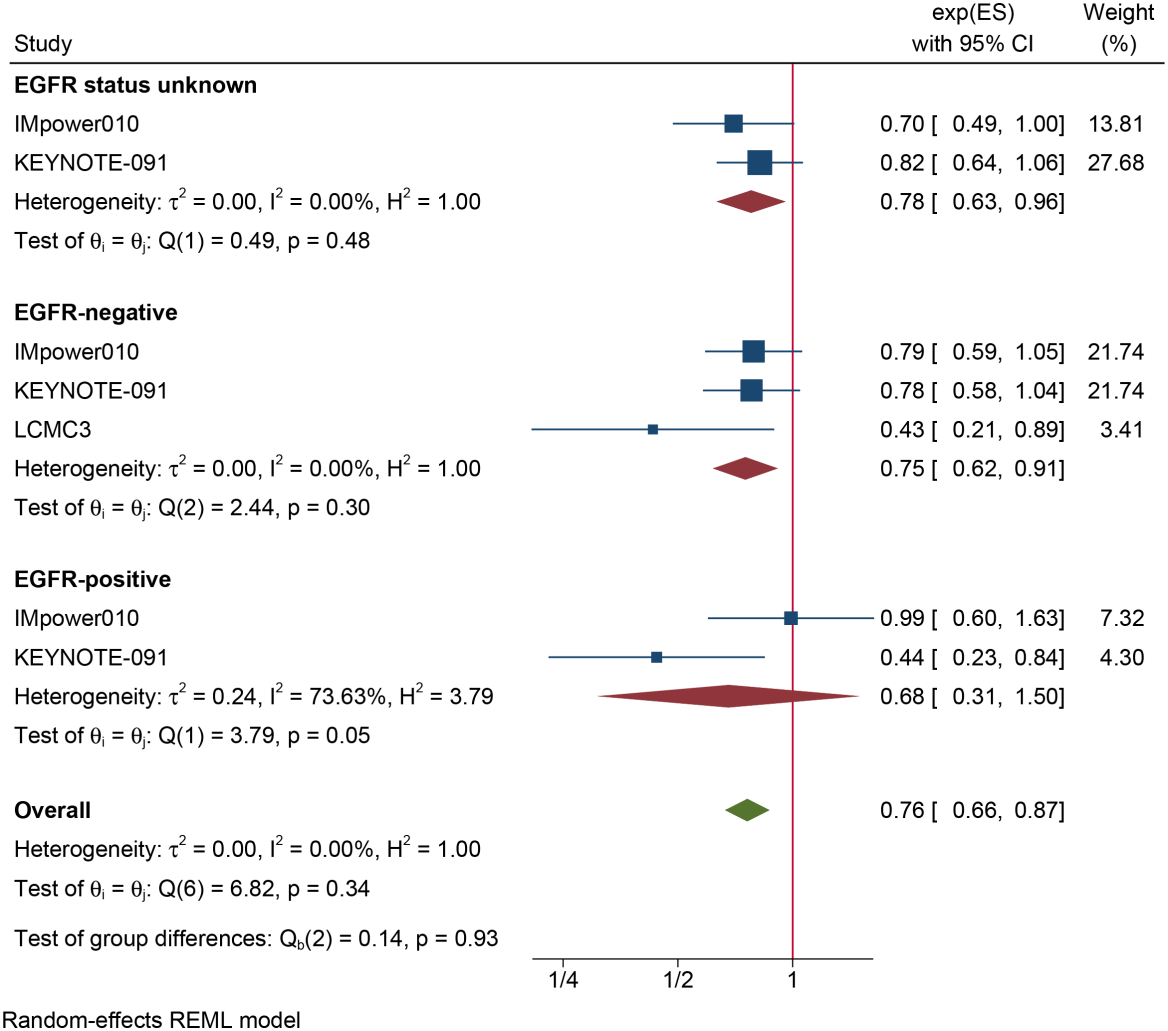
Forest plot of disease-free survival of subgroup analysis based on the type of EGFR mutation.

**Figure 3:**
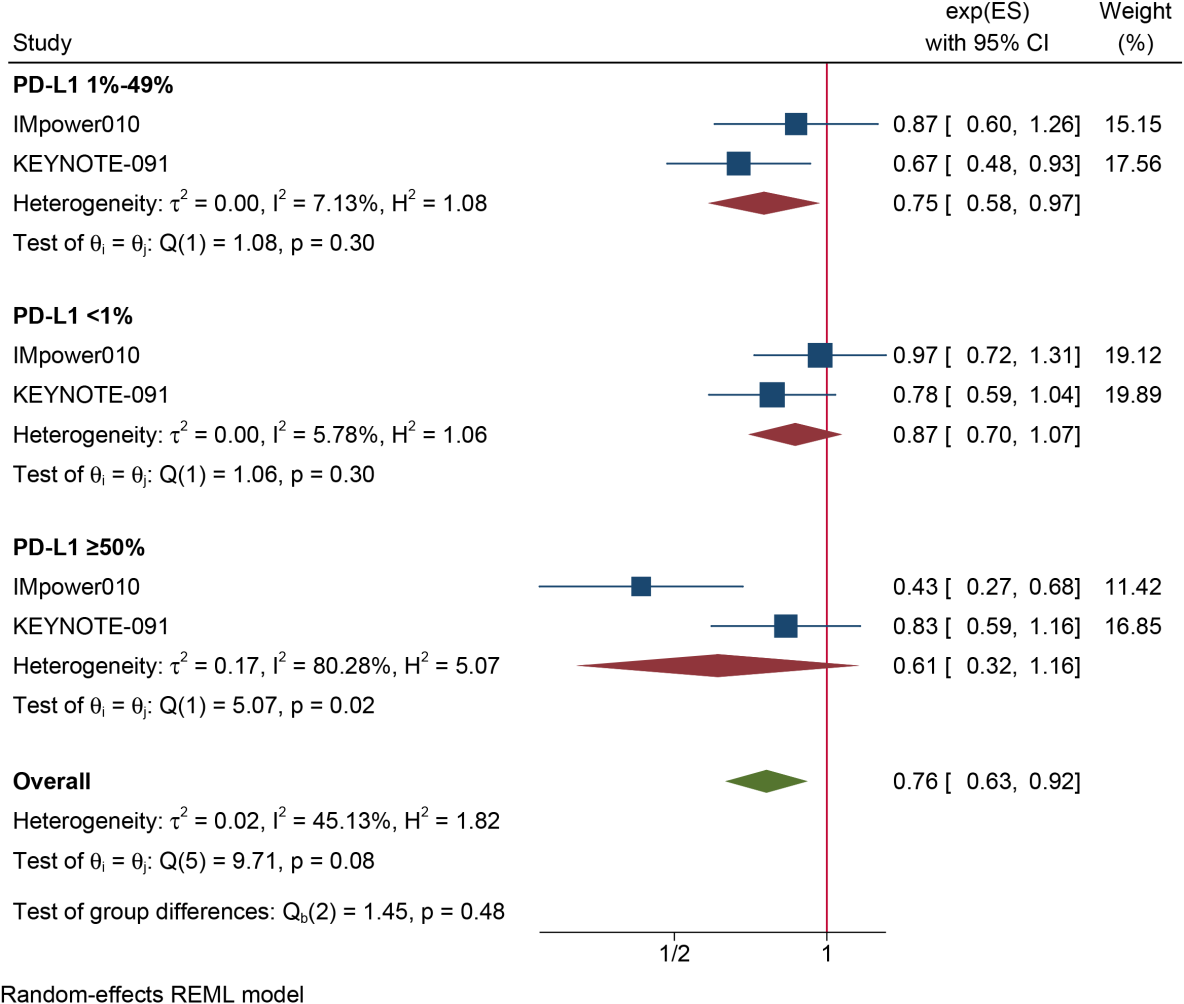
Forest plot of disease-free survival of subgroup analysis based on PD-L1 status.

**Figure 4:**
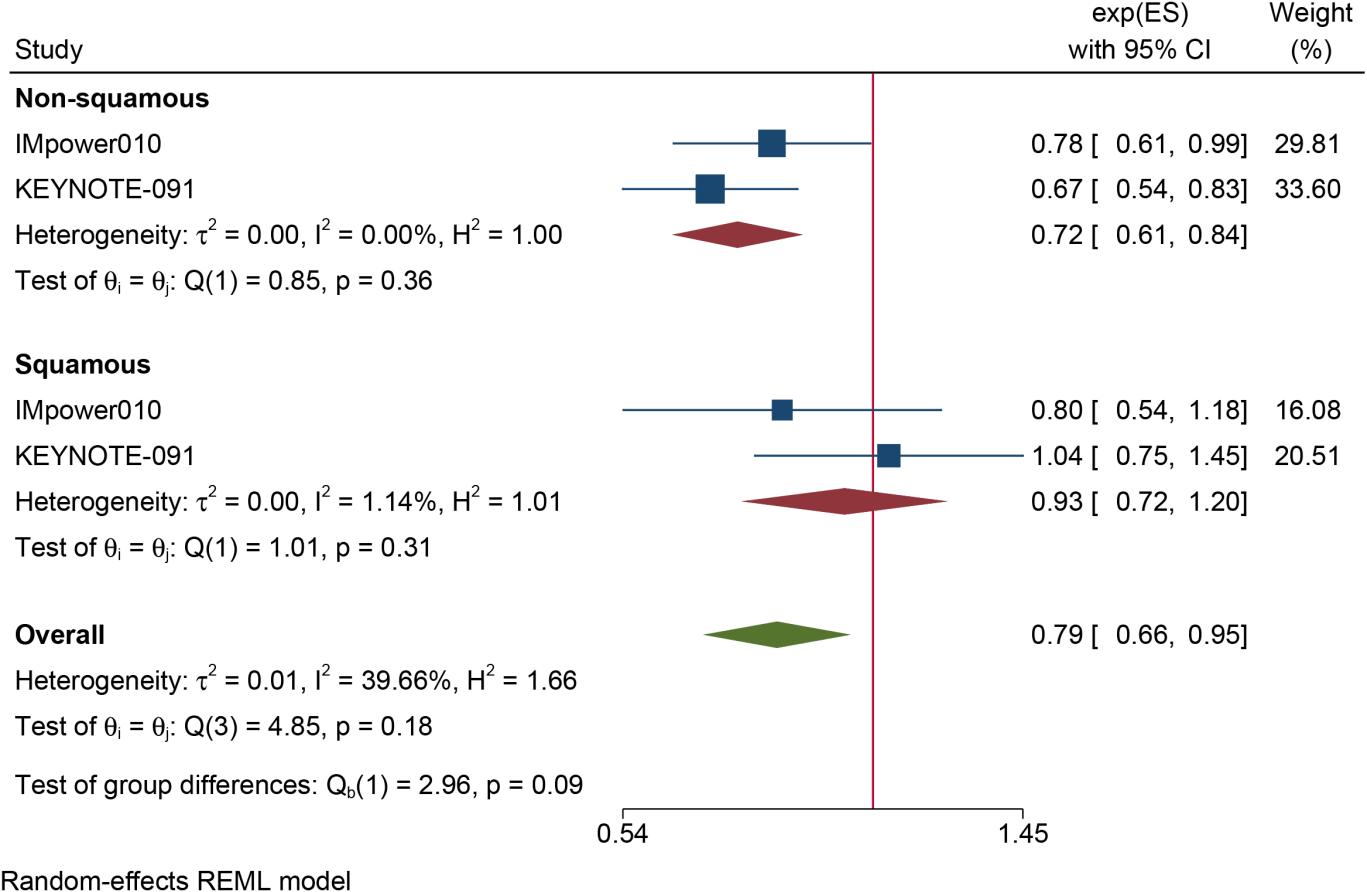
Forest plot of disease-free survival of subgroup analysis based on the histology.

**Figure 5:**
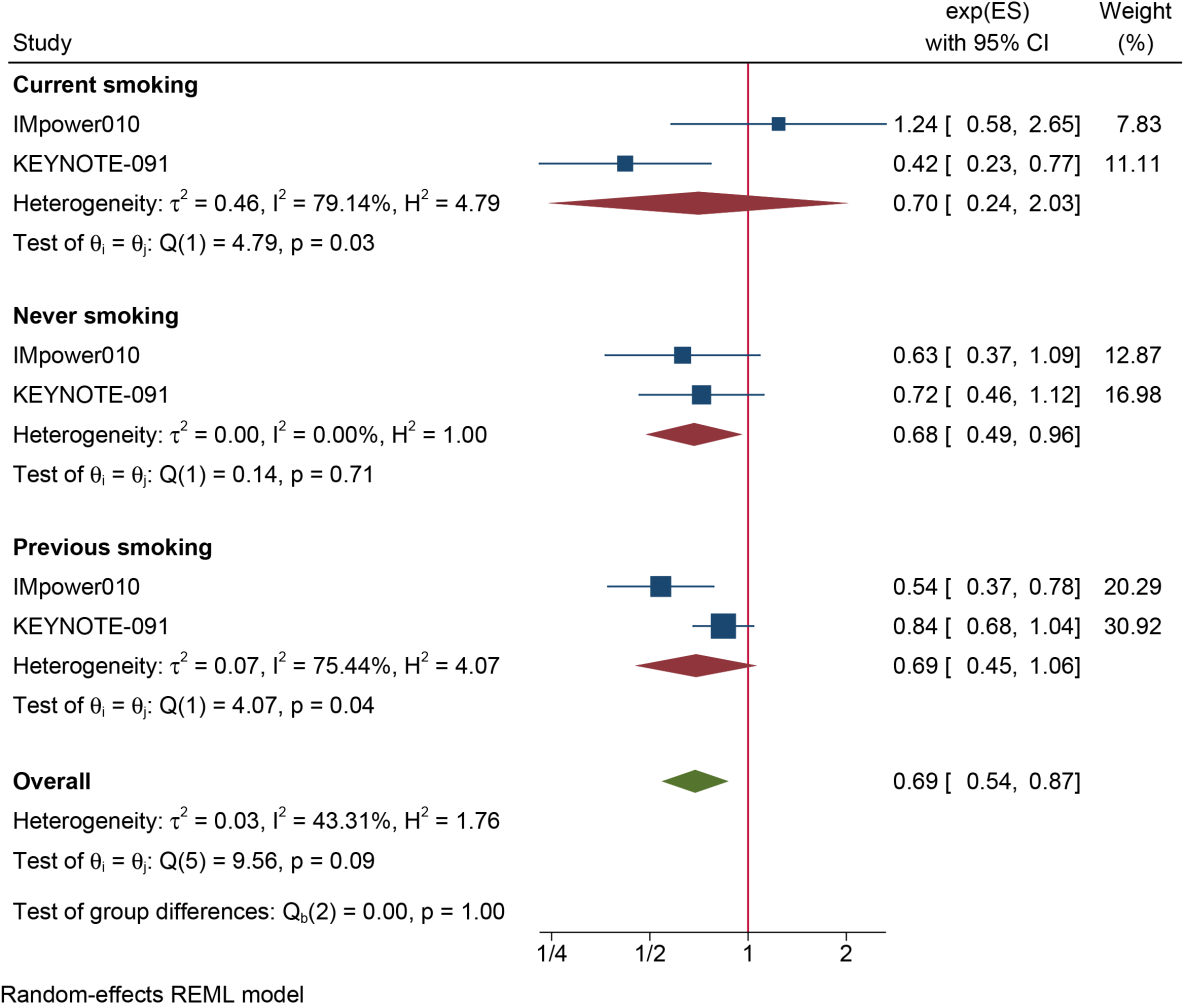
Forest plot of disease-free survival of subgroup analysis based on the smoking status.

The results of the subgroup analysis are as follows: (1) DFS was significantly improved by adjuvant immunotherapy in patients who were EGFR-negative (HR: 0.75, 95% CI: 0.62–0.91, I^2^ = 0%) and in those with unknown EGFR status (HR 0.78, 95% CI: 0.63–0.96, I^2^ = 0%) but not in those who were EGFR-positive (HR: 0.68, 95% CI: 0.31–1.5, I^2^ = 73.6%) (see **Figure 2**). (2) As shown in **Figure 3**, DFS benefit was statistically significant in patients with PD-L1 expression of 1–49% (HR: 0.75, 95% CI: 0.58–0.97, I^2^ = 7.13%) but not in those who were PD-L1-negative (HR: 0.87, 95% CI: 0.70–1.07, I^2^ = 5.78%) or those with PD-L1 ≥50% (HR: 0.61, 95% CI: 0. 32–1.16, I^2^ = 80.28%). (3) Similarly, DFS benefit was significant in patients with non-squamous cell carcinoma (HR: 0.72, 95% CI: 0.61–0.84, I^2^ = 0%) but not in those with squamous cell carcinoma (HR: 0.93, 95% CI: 0.72–1.20, I^2^ = 1.14%) (see **Figure 4**). (4) Notably, adjuvant immunotherapy demonstrated a statistically significant DFS benefit in never-smoking patients (HR: 0.68, 95% CI: 0.49–0.96, I^2^ = 0%). However, neither previous nor current smokers experienced a significant DFS benefit from immunotherapy (**Figure 5**). The aggregated findings are detailed in **Table 2**.

**Table 2:** Summary of the outcomes.

Outcomes	No. of studies	No. of patients	HR (95% CIs) or Rate (95% CIs)	*P*-value	Heterogeneity test	
					I^2^	*P*-value
DFS						
Overall	6	3974	0.82(0.72–0.93)	0.01	0%	0.46
Type of EGFR mutation						
EGFR-positive	2	182	0.68(0.31–1.50)	0.338	73.63%	0.05
EGFR status unknown	2	980	0.78(0.63–0.96)	0.018	0.0%	0.48
EGFR-negative	3	1034	0.75(0.62–0.91)	0.004	0.0%	0.30
PD-L1 status						
PD-L1 <1%	2	848	0.87(0.70–1.07)	0.187	5.78%	0.30
PD-L1 1%-49%	2	626	0.75(0.58–0.97)	0.028	7.13%	0.30
PD-L1 ≥50%	2	562	0.61(0.32–1.16)	0.131	80.20%	0.02
Tumor histology						
Squamous	2	710	0.93(0.72–1.20)	0.588	1.14%	0.31
Non-squamous	2	1349	0.72(0.61–0.84)	<0.001	0%	0.36
Smoking status						
Never smoking	2	245	0.68(0.49–0.96)	0.029	0%	0.71
Previous smoking	2	1168	0.69(0.45–1.06)	0.094	75.44%	0.04
Current smoking	2	240	0.70(0.24–2.03)	0.516	79.14%	0.03
OS						
Overall	4	2618	0.90(0.67–1.21)	0.034	0%	0.37
Rate of TRAEs						
Overall	5	2042	70% (62%–77%)	<0.001	63.45%	0.02
Rate of SAEs						
Overall	6	1416	12% (8%–16%)	<0.001	58.77%	0.06
Rate of discontinuation due to TRAEs						
Overall	5	1414	17% (15%–19%)	<0.001	0.04%	0.64

DFS, disease-free survival; OS, overall survival; EGFR, epidermal growth factor receptor; PD-L1, programmed cell death ligand 1; TRAEs, treatment-related adverse events; SAEs, severe adverse events.

### 3.3 Safety of adjuvant immunotherapy

**Figure 6:**
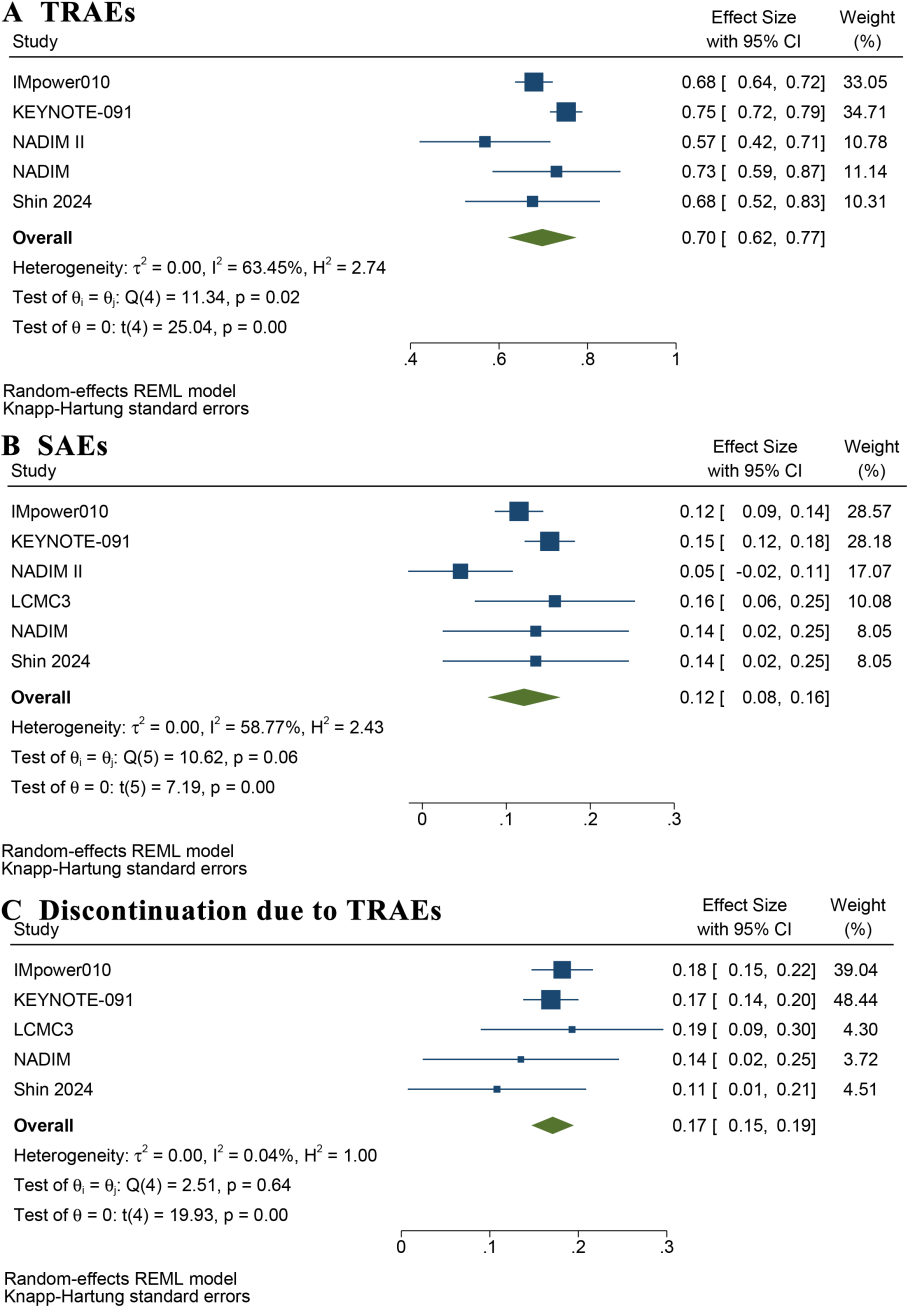
Forest plot of rate of treatment-related adverse events **(A)**, severe adverse events **(B)**, and discontinuation of adjuvant immunotherapy due to treatment-related adverse events **(C)**.

**Supplementary Table S3** summarizes the rates of TRAEs and SAEs reported in these studies. The pooled incidence of TRAEs was 70% (95% CI: 62%–77%) (**Figure 6A**). The estimated incidence of SAEs, defined as grade 3–5 TRAEs, was 12% (95% CI: 8%–16%) (**Figure 6B**). **Figure 6C** shows that the incidence of adjuvant immunotherapy discontinuation due to TRAEs was 17% (95% CI: 15%–19%). A summary of the pooled results is provided in **Table 2**.

**Supplementary Figures S3** and **S4** summarize the profiles of immune-mediated and severe immune-mediated AEs, respectively. Immune-mediated AEs with an incidence >5% included hypothyroidism (19.2%), rash (18.4%), pneumonitis (10.2%), and hyperthyroidism (8.7%). The most prevalent severe immune-mediated AEs included severe skin reactions (1.9%), hepatitis (1.2%), pneumonitis (1.1%), and rashes (0.9%).

### 3.4 Sensitivity analysis and publication bias

Leave-one-out sensitivity analyses indicated that no single study significantly affected the pooled DFS and OS results (see **Supplementary Figure S5**). The pooled HRs for DFS (0.82, 95% CI: 0.74–0.90) and OS (0.90, 95% CI: 0.76–1.08) from the fixed-effects model closely matched those from the random-effects method (see **Supplementary Figure S6**). These results confirm the robustness of our findings. Furthermore, the heterogeneity in TRAE incidence significantly decreased after excluding the KEYNOTE-091 trial (22) (**Supplementary Figure S7**). Similarly, removing the KEYNOTE-091 (22) or NADIM II (28) trials appreciably reduced the heterogeneity in SAE rates (**Supplementary Figure S8**). The funnel plots (**Supplementary Figure S9**), and Egger’s test (**Supplementary Table S4**) showed no significant publication bias.

## 4 Discussion

ICIs have become a critical treatment pillar for advanced-stage NSCLC without oncogenic drivers. Their efficacy as neo/adjuvant or perioperative immunotherapy in resectable NSCLC has been confirmed in previous meta-analysis results see **Supplementary Table S3**). However, no study has specifically summarized the evidence on adjuvant immunotherapy in patients with completely resected IB–III NSCLC.

Nuccio et al. (34) conducted a meta-analysis by examining clinicopathological factors influencing ICI benefits in early-stage lung cancer and comparing neoadjuvant and perioperative strategies. Their analysis, which included two RCTs on adjuvant immunotherapy, found that it prolonged DFS. Expanding upon this with updated data and additional studies, we found that adjuvant ICI therapy significantly prolonged DFS—particularly in EGFR-negative, PD-L1 (1–49%), non-squamous carcinoma, or never-smokers—it did not improve OS in completely resected stage IB–III NSCLC. These findings have clinical implications for selecting adjuvant therapy in this patient population.

It is extensively accepted that upregulated PD-L1 expression in tumor cells plays an essential role in tumor immune evasion. Specifically, PD-L1 binding to PD-1 inhibits T cell proliferation and survival through the PI3K/Akt pathway and disrupts T cell differentiation by blocking the synthesis and secretion (35, 36). Additionally, PD1/PD-L1 signaling inhibits T cell function by attenuating CD28 costimulatory signaling (37).

Consequently, the PD-1/PD-L1 blockade therapy, which directly restores suppressed host antitumor immune responses, has significantly improved the clinical prognosis of many patients with advanced and resectable NSCLC (38–40). Our study further confirms the DFS benefits of adjuvant ICI therapy in patients who have undergone complete resection. The non-significant OS benefit may occur because the median OS in the intention-to-treat (ITT) population has yet to be reached in two large RCT studies. Another important consideration is that none of the included studies identified OS as the primary endpoint, which may have resulted in insufficient power to detect statistically significant differences in OS (41).

PD-L1 expression in tumors is often used to select suitable candidates for immunotherapy. It is generally believed that the higher the expression of PD-L1, the better the therapeutic effect of immunotherapy. Indeed, the study in 2021 by Felip et al. (15) found that DFS has significantly improved in the PD-L1 ≥50% population but not in the PD-L1 of 1–49% and PD-L1<1% populations, based on data from 882 stage II to IIIA patients followed 32.2 months (IQR 27.4–38.3). However, the other RCT in 2022 (22), which included 1177 stage IB to IIIA patients, followed at 35.6 months (IQR 27.1–45.5), indicating the significant DFS benefit in the PD-L1 of 1–49% patients, but not in the PD-L1 ≥50% and PD-L1 <1% populations. The investigators considered that random factors contributed to the better-than-expected DFS performance in the control group of patients with PD-L1 ≥50%, and further extended follow-up periods were needed to identify the survival advantage of the experimental group. Nevertheless, the final DFS analysis, based on the data from 51.7 months (range 32.7–84.2) follow-up, indicated the same result (17). Our pooled results further showed that patients with PD-L1 of 1–49% benefited from postoperative immunotherapy, whereas those with PD-L1 expression ≥50% did not. These findings suggest we should be cautious when applying postoperative immunotherapy in patients with PD-L1 expression ≥ 50%. More RCT trials are required to support and verify these results.

Our findings indicate that DFS benefits were observed only in the never-smoker population. Given that smoking increases the tumor mutation burden (TMB) in tumor cells (42), smokers tend to benefit from immunotherapy than never-smokers. However, the effect of smoking on immunotherapy efficacy in advanced NSCLC has been inconsistent across clinical strategies. In first-line treatment, smokers benefit more from immunotherapy alone than never-smokers (43). Conversely, when first-line chemoimmunotherapy is used, both smoking and nonsmoking subgroups experience similar clinical benefits (44).

The efficacy of different immunotherapeutic agents in NSCLC also varies by smoking status. A meta-analysis (45) demonstrated that both smokers and never-smokers with NSCLC gained significant OS benefits from pembrolizumab plus chemotherapy, with a reduced risk of death of 32–46% in smokers and 70–84% in never-smokers, respectively. However, only smokers benefited from atezolizumab combined with chemotherapy. These differences may stem from variations in the pathological molecular characteristics and immune microenvironments of the tumors. Filetti et al. (46) conducted a retrospective analysis of 142 patients with PD-L1 expression >50% who received first-line pembrolizumab monotherapy. Their results showed that among patients with high TMB, never-smokers exhibited a higher overall response rate (100% vs. 73%) and longer median OS (27.95 months vs. 17.65 months) than smokers. Further analysis suggested that this difference was caused by the enrichment of DNA damage response signaling pathways in the tumor tissues of never-smokers.

The superior DFS benefits observed in never-smokers in our meta-analysis may be attributed to similar tumor molecular characteristics. We plan to further verify these findings in future clinical studies. Additionally, given the limited number of studies included in the subgroup analysis and the high heterogeneity in both the previous and current smoking subgroups, the conclusion that DFS benefits were absent in these two populations should be interpreted cautiously.

Our subgroup analysis indicated that adjuvant immunotherapy significantly improved DFS in patients with resected EGFR-negative NSCLC and non-squamous cell carcinoma. Consistent with our findings, previous studies indicate that patients with advanced NSCLC harboring EGFR mutations derive limited benefit from immunotherapy (47–49). Most clinical studies have shown that tumors with EGFR mutations exhibit lower PD-L1 expression than those with wild-type EGFR (50–52). Conversely, wild-type EGFR tumors are characterized by higher TMB (53), stronger T-cell clonality (54), and significant infiltration of CD8+ T cells (53), all of which contribute to enhanced immunotherapy efficacy. Future studies are needed to validate the efficacy across different histological types and molecular subgroups of NSCLC.

This meta-analysis had several limitations. First, the number of trials enrolled in this study was relatively small, and the inclusion of some single-arm trials and retrospective cohort studies may have influenced the overall level of evidence. However, our findings were based on data from 4048 patients, and the quality of the included studies was acceptable, with no significant bias. Additionally, the OS and safety outcomes were consistent with those of previous studies. Second, the OS data were derived from an interim analysis, as the follow-up endpoints of large RCTs had not yet been reached. A longer follow-up period is needed to determine whether a significant improvement in the OS of the ITT population exists. Third, among the included studies, patients in the NADIM II, NADIM, and Shin 2024 trials only received adjuvant immunotherapy alone. In the NeoR-World, KEYNOTE-091, and IMpower010 trials, 84.8% to 100% of participants additionally underwent 1-4 cycles of adjuvant chemotherapy. In the BR.31 trial, patients were permitted to receive adjuvant chemotherapy, while in the LCMC3 trial, adjuvant chemotherapy with or without radiotherapy was allowed, but data on combination therapy were unclear. We intended to perform a subgroup analysis to evaluate the confounding effects of chemotherapy combination therapy. However, insufficient data prevented this execution.

Overall, our findings suggest that adjuvant immunotherapy improves DFS in patients with resected NSCLC, particularly in those who are EGFR-negative, have PD-L1 expression of 1–49%, have non-squamous cell carcinoma, and have never smoked. This treatment regimen demonstrates a favorable safety and tolerability profile. These results highlight adjuvant immunotherapy as a promising therapeutic option for IB–III NSCLC following complete resection. Further clinical trials are warranted to clarify its role across different NSCLC stages.

## Data Availability

The original contributions presented in the study are included in the article/**Supplementary Material**. Further inquiries can be directed to the corresponding author.
