# Supplementary material for: Adjuvant immunotherapy improves survival in completely resected stage IB–III NSCLC: a systematic review and meta-analysis
Source: Front Oncol. 2025 Apr 8;15:1493221. doi: 10.3389/fonc.2025.1493221 (PMC12011596; doi:10.3389/fonc.2025.1493221)
Supplement: Supplementary file 1 [file DataSheet1.docx]

Supplementary Material

**Figure legends**

**Figure. S1** The protocol for searching and selecting studies

**Figure. S2** Summary of bias risk within included RCTs by Cochrane Collaboration

**Figure. S3** Pooled incidence of immune-mediated adverse events reported in clinical trials

**Figure. S4** Pooled incidence of severe immune-mediated adverse events reported in clinical trials

**Figure. S5** Sensitivity analysis of DFS(A), and OS(B)

**Figure. S6** Sensitivity analysis of DFS(A), and OS(B) by using a fixed effects model

**Figure. S7** Sensitivity analysis of treatment-related adverse events

**Figure. S8** Sensitivity analysis of severe adverse events

**Figure. S9** Funnel plots


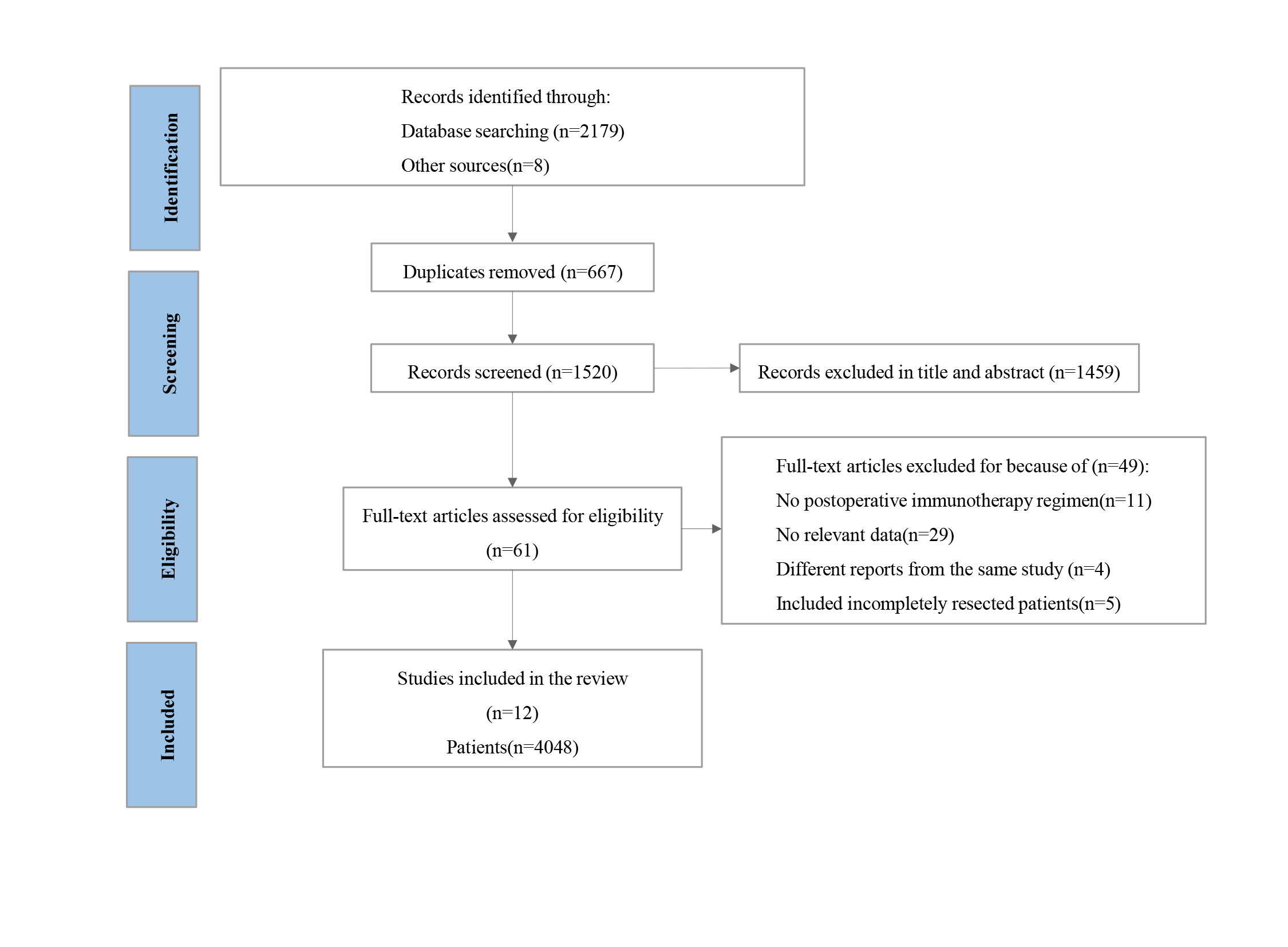


**Figure. S1** The protocol for searching and selecting studies


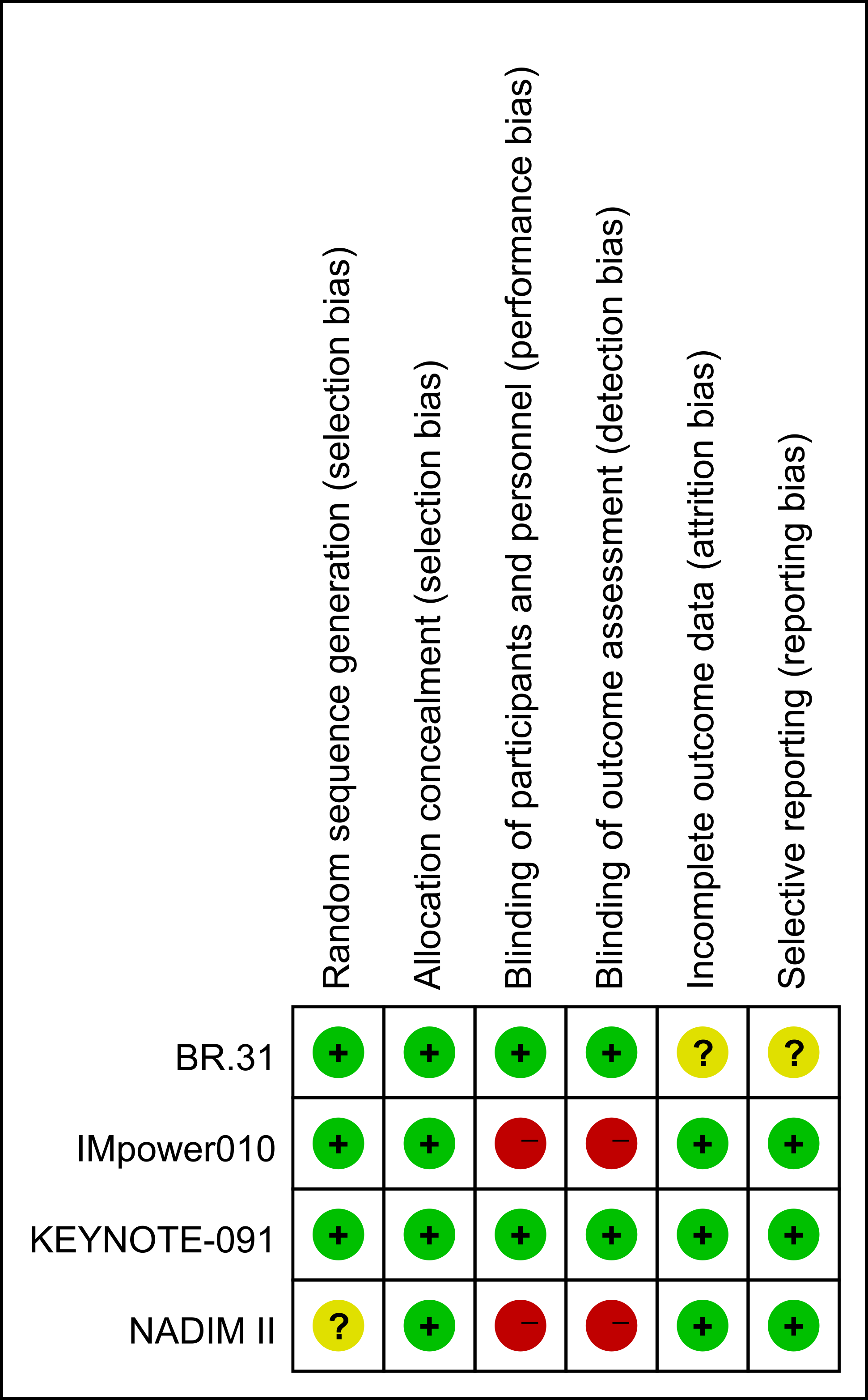


**Figure. S2** Summary of bias risk within included RCTs by Cochrane Collaboration


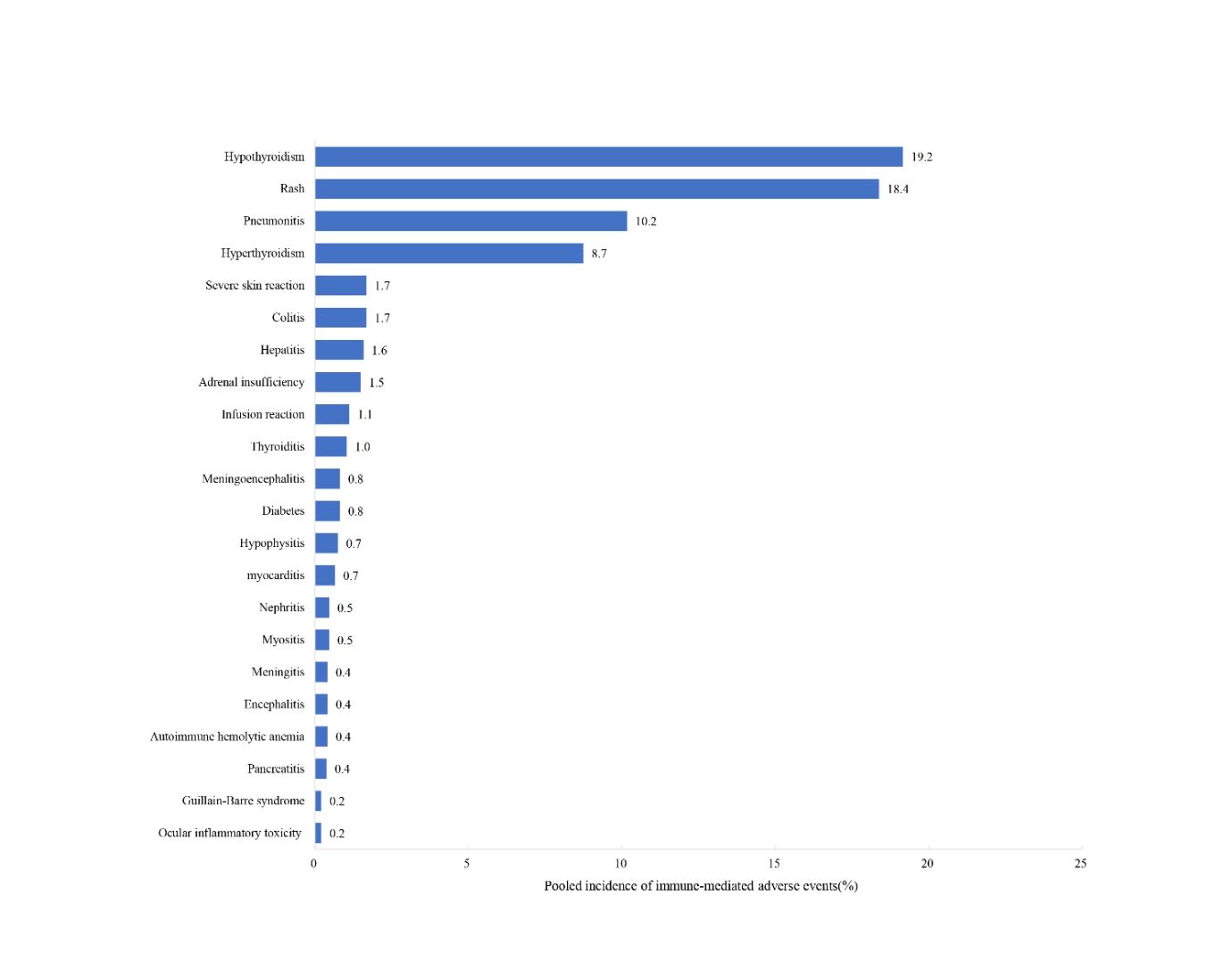


**Figure. S3** Pooled incidence of immune-mediated adverse events reported in clinical trials


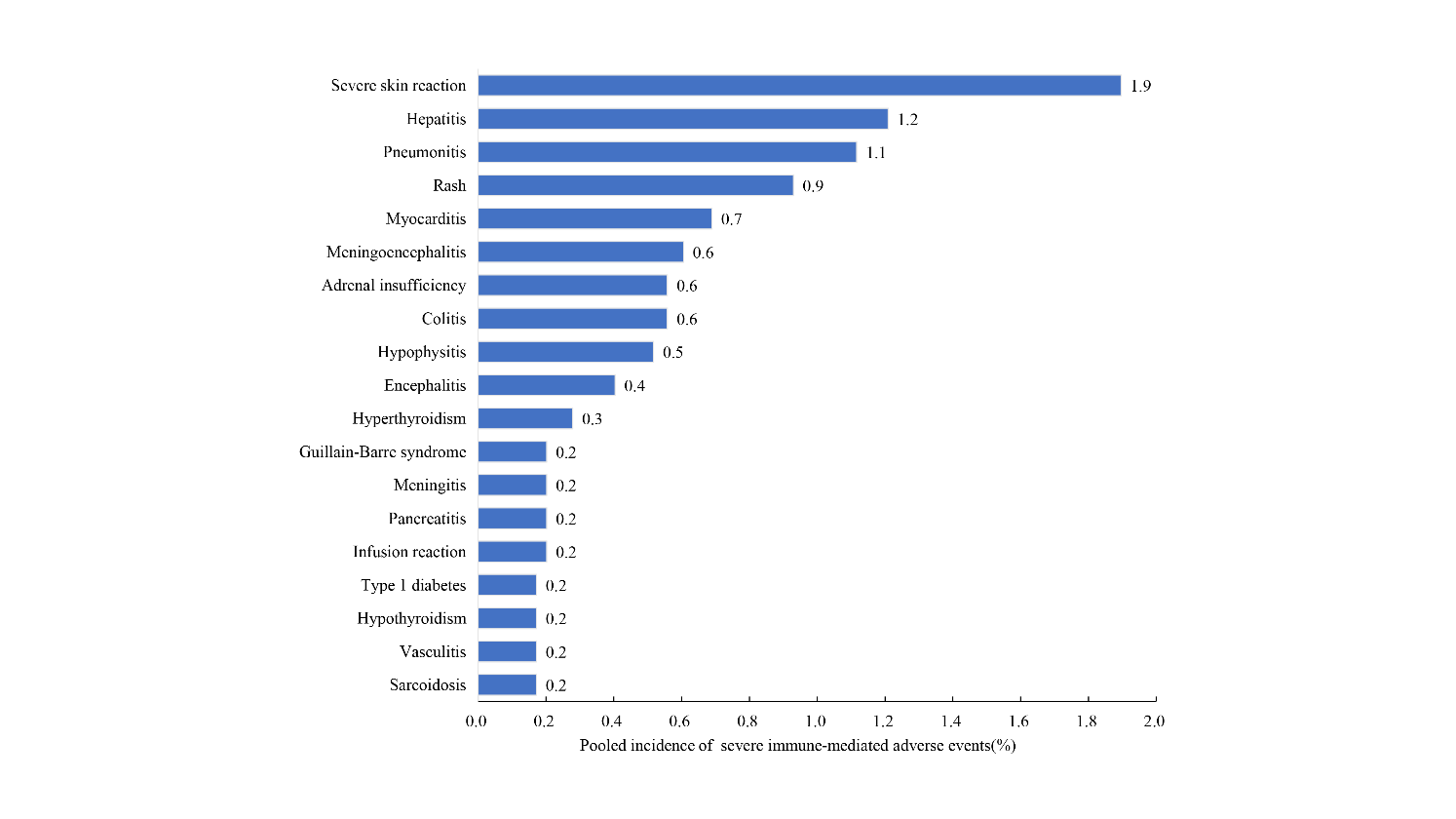


**Figure. S4** Pooled incidence of severe immune-mediated adverse events reported in clinical trials


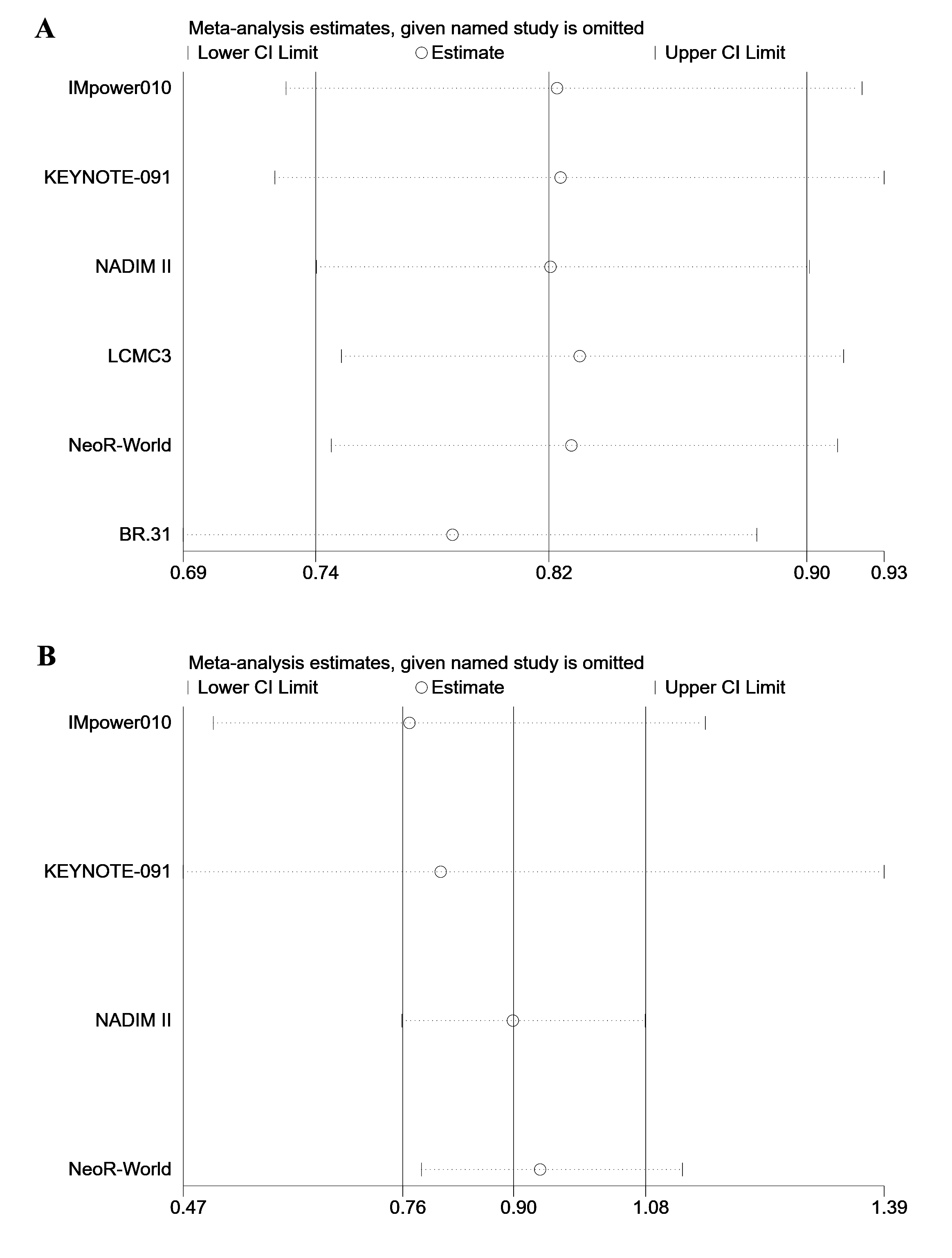


**Figure. S5** Sensitivity analysis of DFS(A), and OS(B)


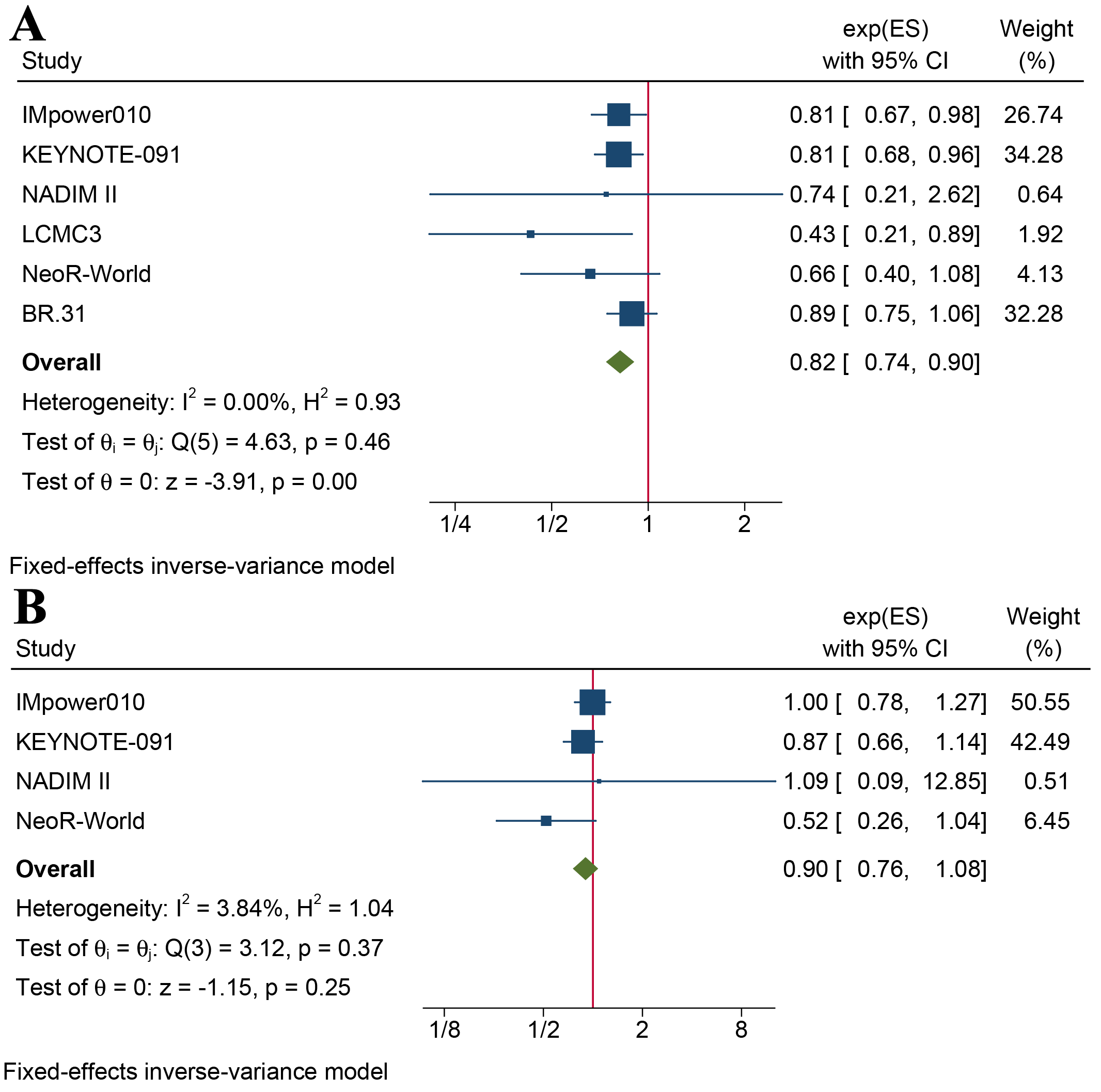


**Figure. S6** Sensitivity analysis of DFS(A), and OS(B) by using a fixed effects model


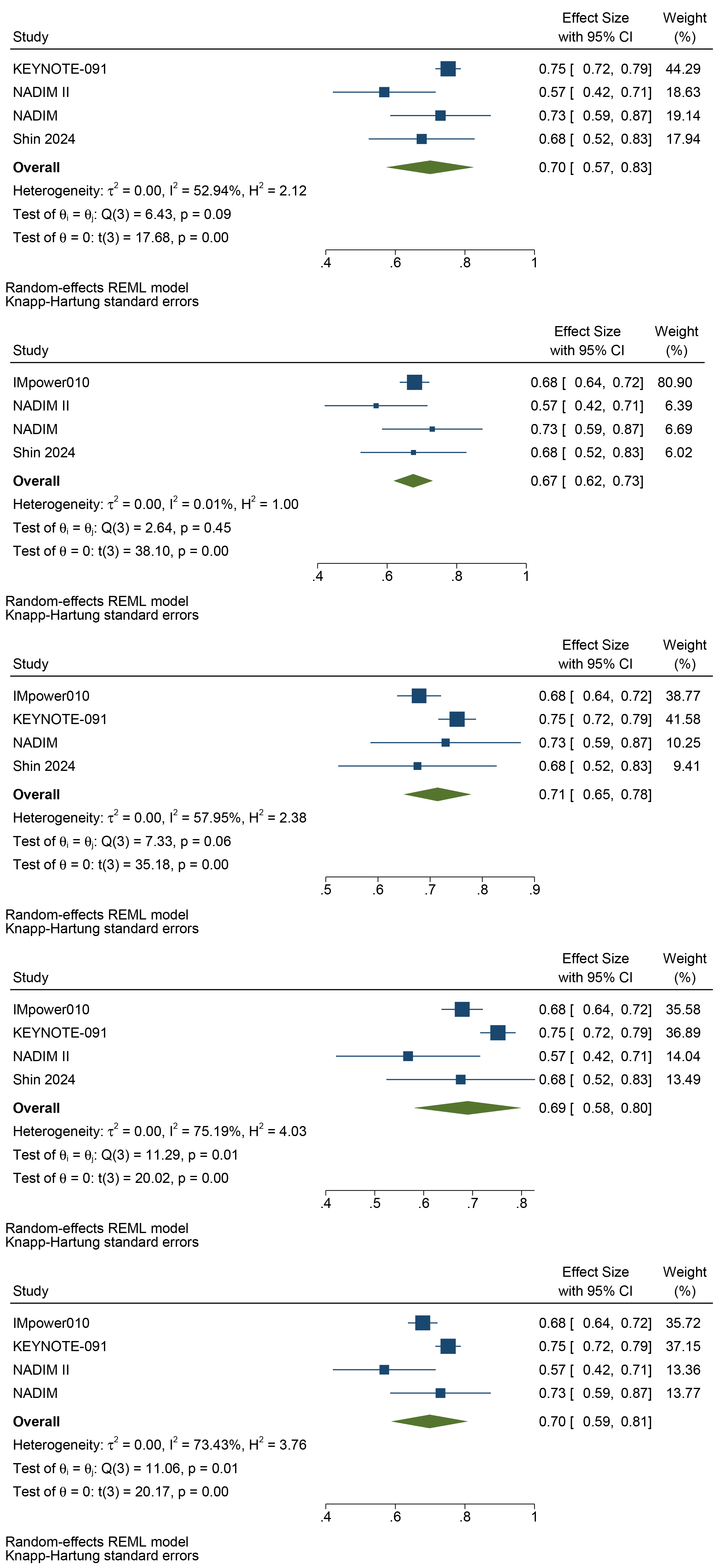


**Figure. S7** Sensitivity analysis of treatment-related adverse events.


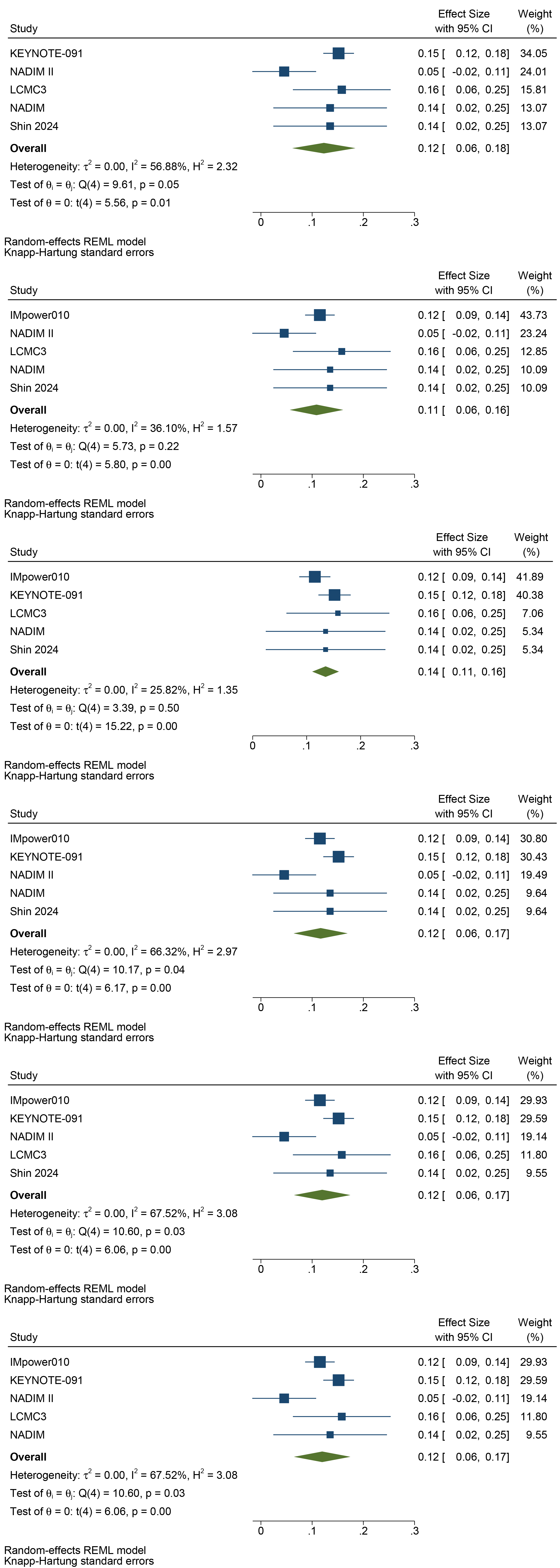


**Figure. S8** Sensitivity analysis of severe adverse events.


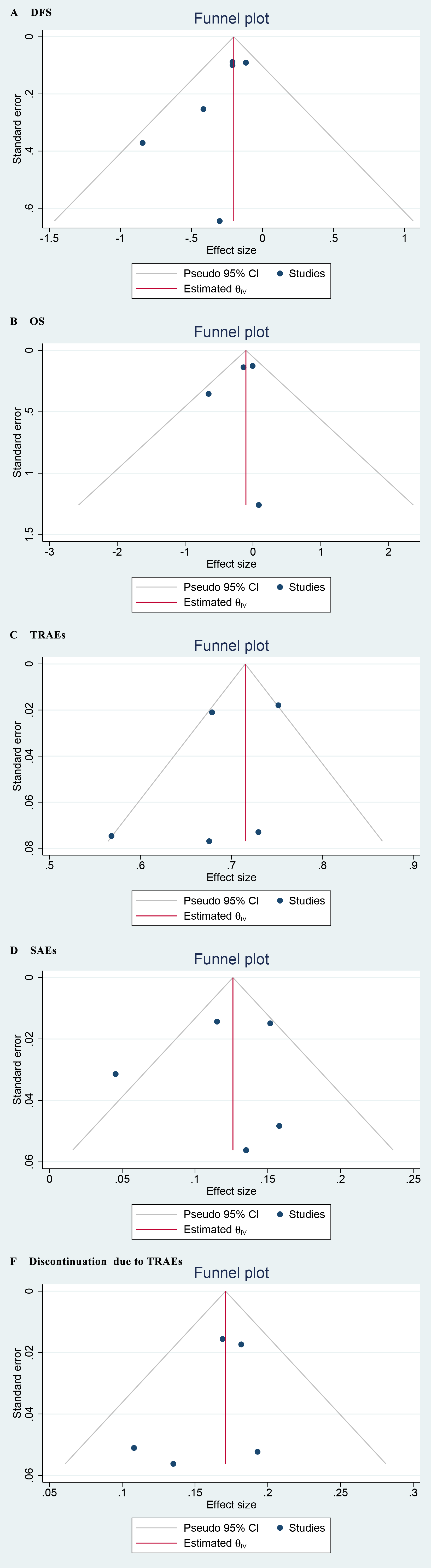


**Figure. S9** Funnel plots.

**Supplementary Table S1.** PRISMA guidelines checklist

| **Section/topic** | **#** | **Checklist item** | **Reported on page #** |
| --- | --- | --- | --- |
| **TITLE** | | |  |
| Title | 1 | Identify the report as a systematic review, meta-analysis, or both. | 1 |
| **ABSTRACT** | | |  |
| Structured summary | 2 | Provide a structured summary including, as applicable: background; objectives; data sources; study eligibility criteria, participants, and interventions; study appraisal and synthesis methods; results; limitations; conclusions and implications of key findings; systematic review registration number. | 2-3 |
| **INTRODUCTION** | | |  |
| Rationale | 3 | Describe the rationale for the review in the context of what is already known. | 2-3 |
| Objectives | 4 | Provide an explicit statement of questions being addressed with reference to participants, interventions, comparisons, outcomes, and study design (PICOS). | 2-3 |
| **METHODS** | | |  |
| Protocol and registration | 5 | Indicate if a review protocol exists, if and where it can be accessed (e.g., Web address), and, if available, provide registration information including registration number. | 3 |
| Eligibility criteria | 6 | Specify study characteristics (e.g., PICOS, length of follow-up) and report characteristics (e.g., years considered, language, publication status) used as criteria for eligibility, giving rationale. | 3 |
| Information sources | 7 | Describe all information sources (e.g., databases with dates of coverage, contact with study authors to identify additional studies) in the search and date last searched. | 3 |
| Search | 8 | Present full electronic search strategy for at least one database, including any limits used, such that it could be repeated. | 3 |
| Study selection | 9 | State the process for selecting studies (i.e., screening, eligibility, included in systematic review, and, if applicable, included in the meta-analysis). | 3 |
| Data collection process | 10 | Describe method of data extraction from reports (e.g., piloted forms, independently, in duplicate) and any processes for obtaining and confirming data from investigators. | 3 |
| Data items | 11 | List and define all variables for which data were sought (e.g., PICOS, funding sources) and any assumptions and simplifications made. | 3 |
| Risk of bias in individual studies | 12 | Describe methods used for assessing risk of bias of individual studies (including specification of whether this was done at the study or outcome level), and how this information is to be used in any data synthesis. | 3 |
| Summary measures | 13 | State the principal summary measures (e.g., risk ratio, difference in means). | 3-4 |
| Synthesis of results | 14 | Describe the methods of handling data and combining results of studies, if done, including measures of consistency (e.g., 2) for each meta-analysis. | 3-4 |
| **Section/topic** | **#** | **Checklist item** | **Reported on page #** |
| Risk of bias across studies | 15 | Specify any assessment of risk of bias that may affect the cumulative evidence (e.g., publication bias, selective reporting within studies). | 5 |
| Additional analyses | 16 | Describe methods of additional analyses (e.g., sensitivity or subgroup analyses, meta-regression), if done, indicating which were pre-specified. | 5 |
| **RESULTS** | | |  |
| Study selection | 17 | Give numbers of studies screened, assessed for eligibility, and included in the review, with reasons for exclusions at each stage, ideally with a flow diagram. | 4 |
| Study characteristics | 18 | For each study, present characteristics for which data were extracted (e.g., study size, PICOS, follow-up period) and provide the citations. | 4 |
| Risk of bias within studies | 19 | Present data on risk of bias of each study and, if available, any outcome level assessment (see item 12). | 4-5 |
| Results of individual studies | 20 | For all outcomes considered (benefits or harms), present, for each study: (a) simple summary data for each intervention group (b) effect estimates and confidence intervals, ideally with a forest plot. | 4 |
| Synthesis of results | 21 | Present results of each meta-analysis done, including confidence intervals and measures of consistency. | 4-5 |
| Risk of bias across studies | 22 | Present results of any assessment of risk of bias across studies (see Item 15). | 5 |
| Additional analysis | 23 | Give results of additional analyses, if done (e.g., sensitivity or subgroup analyses, meta-regression [see Item 16]). | 4-5 |
| **DISCUSSION** | | |  |
| Summary of evidence | 24 | Summarize the main findings including the strength of evidence for each main outcome; consider their relevance to key groups (e.g., healthcare providers, users, and policy makers). | 5-7 |
| Limitations | 25 | Discuss limitations at study and outcome level (e.g., risk of bias), and at review-level (e.g., incomplete retrieval of identified research, reporting bias). | 7 |
| Conclusions | 26 | Provide a general interpretation of the results in the context of other evidence, and implications for future research. | 7 |
| **FUNDING** | | |  |
| Funding | 27 | Describe sources of funding for the systematic review and other support (e.g., supply of data), role of funders for the systematic review. | 8 |

**Supplementary Table S2.** Summary of bias risk within included non-RCTs by non-randomized studies of interventions tool.

| Study | Bias due to confounding | Bias in selection of participants | Bias in classification of interventions | Bias due to deviations from intended interventions | Bias due to missing data | Bias in measurement of outcomes | Bias in selection of the reported results | Overall |
| --- | --- | --- | --- | --- | --- | --- | --- | --- |
| Chaft 2022 Carbone 2023 | No information | Low risk | Low risk | Low risk | Low risk | Low risk | Low risk | Low risk |
| Yang 2024 | No information | Low risk | Low risk | Low risk | Low risk | Low risk | Low risk | Low risk |
| Provencio 2020 Provencio 2022 | No information | Low risk | Low risk | Low risk | Low risk | Low risk | Low risk | Low risk |
| Shin 2024 | No information | Low risk | Low risk | Low risk | Low risk | Low risk | Low risk | Low risk |

**Supplementary Table S3.** Research data on the endpoints in clinical trials

| Study | Clinical trial | Follow-up | HR for DFS | HR for OS | Incidence of TRAEs | Incidence of SAEs | Incidence of discontinuation due to TRAEs |
| --- | --- | --- | --- | --- | --- | --- | --- |
| Felip  2021 (15)  Felip  2023 (14) | IMpower010 | 45.3(35.5–52.3) | 0.81(0.67–0.99) | 0.99(0.78–1.28) | 68(336/495) | 12(57/495) | 18(90/495) |
| O'Brien  2022 (21)  Besse  2023 (17) | KEYNOTE-091 | 51.7 (32.7–84.2) | 0.81(0.68–0.96) | 0.87(0.67–1.15) | 75(436/580) | 15(88/580) | 17(98/580) |
| Provencio 2023 (28) | NADIM II | 21.6(17.4–30.9) | 0.74(0.21–2.63) | 1.09(0.09–12.5) | 57(25/44) | 5(2/44) | - |
| Chaft  2022 (31)  Carbone  2023 (32) | LCMC3 | 36 | 0.43(0.21–0.9) | - | - | 16(9/57) | 19(11/57) |
| Yang  2024 (16) | NeoR-World | - | 0.66(0.4–1.08) | 0.52(0.26–1.04) | - | - | - |
| Provencio  2020 (30)  Provencio  2022 (29) | NADIM | 38 (36.7–40.7) | - | - | 73(27/37) | 14(5/37) | 14(5/37) |
| Shin  2024 (23) | - | 71 | - | - | 68(25/37) | 14(5/37) | 11(4/37) |
| Goss  2024 (33) | BR.31 | 60 | 0.89 (0.75-1.07) | - | - | - | - |

DFS, disease-free survival; OS, overall survival; TRAEs, treatment-related adverse events; SAEs, severe adverse events.

**Supplementary Table S4.** *P* value of Egger's test.

|  | DFS | OS | TRAEs | SAEs | Discontinuation due to AEs |
| --- | --- | --- | --- | --- | --- |
| *P* | 0.103 | 0.409 | 0.412 | 0.771 | 0.393 |

DFS, disease-free survival, OS, overall survival, TRAEs, treatment-related adverse events, SAEs, severe adverse events

**Supplementary Table S5** Characteristics of meta-analyses on neo/adjuvant or perioperative immunotherapy for NSCLC published within the last 3 years

| Author/year | Aim | No. of studies | Types of included studies | No. of patients | Main outcomes | Conclusion |
| --- | --- | --- | --- | --- | --- | --- |
|  |  |  |  |  |  |  |
| Zhang et al. 2024 (1) | To evaluate the efficacy and safety of perioperative immunotherapy combined with chemotherapy in resectable NSCLC. | 5 | RCTs | 2912 | OS, EFS, pCR, MPR, R0 resection rate, and AEs | Perioperative immunotherapy combined with chemotherapy was superior to chemotherapy alone in tumor regression and survival while increasing the risk of TRAEs. |
| Li et al. 2023 (2) | To compare the relative efficacy of adjuvant EGFR-TKI with immunotherapy in completely resected, EGFR-mutant NSCLC. | 7 | RCTs | 1471 | DFS | In patients with EGFR mutation, adjuvant osimertinib has better efficacy than chemotherapy plus immunotherapy. |
| Zhou et al. 2024 (3) | To compare the efficacy and safety of neoadjuvant-adjuvant immunotherapy with neoadjuvant-only immunotherapy in resectable NSCLC. | 5 | Randomized prospective studies | 2385 | EFS, OS, and TRAEs | Compared to neoadjuvant-only immunotherapy, neoadjuvant-adjuvant immunotherapy did not improve survival for patients with resectable NSCLC and was associated with a higher incidence of TRAEs. |
| Nuccio et al. 2023 (4) | To identify the factors influencing the efficacy of ICIs in early-stage NSCLC and to compare perioperative with neoadjuvant strategies. | 8 | RCTs | 4661 | pCR, EFS, DFS, MPR, OS, toxicity, and surgical outcomes | PD-L1 status, pCR, and stage impact the survival outcomes of patients treated with neoadjuvant or perioperative ICIs. Regarding survival outcomes, neoadjuvant therapy alone appears to be comparable to the perioperative ICI plus chemotherapy strategy. |
| Han et al. 2024 (5) | To indirectly compare the efficiency of neoadjuvant and neoadjuvant plus adjuvant immunotherapy in resectable NSCLC. | 8 | RCTs | 3429 | EFS, OS, pCR, MPR, and ORR | For patients with PD-L1 < 1% or 1-49%, neoadjuvant plus adjuvant immunotherapy and neoadjuvant immunotherapy had similar EFS. However, patients with PD-L1≥50% may obtain more EFS benefits from neoadjuvant immunotherapy than neoadjuvant plus adjuvant immunotherapy. |
| Chen et al. 2023 (6) | To evaluate the validity of MPR as a predictor of survival after neoadjuvant immunotherapy in resectable NSCLC. | 53 | RCTs, prospective nonrandomized and retrospective studies | 2807 | MPR and PCR | Neoadjuvant chemo-immunotherapy leads to a higher MPR in NSCLC patients. MPR can be utilized as a surrogate marker to evaluate the effectiveness of neoadjuvant immunotherapy. |
| Pasqualotto et al. 2023 (7) | To compare the efficacy and safety of neoadjuvant or adjuvant immunotherapy plus chemotherapy with chemotherapy alone in resectable NSCLC. | 7 | RCTs | 3915 | OS, DFS, MPR, pCR, and AEs | Neoadjuvant or adjuvant immunotherapy plus chemotherapy can improve patient survival compared to chemotherapy alone. |
| Meng et al. 2024 (8) | To compare the efficacy and safety of neoadjuvant, adjuvant, and perioperative immunotherapies in early-stage NSCLC. | 10 | RCTs | 5569 | pCR, MPR, AEs, irAEs, EFS/DFS, and OS | Perioperative immunotherapy plus chemotherapy seems more effective than neoadjuvant immunotherapy plus chemotherapy and adjuvant immunotherapy. |
| Zhang et al. 2025 (9) | To compare the efficacy and safety of neo/adjuvant and perioperative chemoimmunotherapy with chemotherapy alone in resectable NSCLC. | 13 | RCTs | 6704 | PFS (EFS/DFS), OS, and AEs of grade 3 or greater | Neoadjuvant and perioperative chemoimmunotherapy can improve patient survival compared to standard chemotherapy. |
| Wu et al. 2023 (10) | To analyze the comparative effectiveness of neoadjuvant chemoimmunotherapy versus chemotherapy alone in resectable NSCLC and explore factors predictive of neoadjuvant immunotherapy benefits. | 66 | RCTs, prospective nonrandomized and retrospective studies | 3289 | pCR, DFS, PFS, EFS, RFS, OS, and AEs | Compared to neoadjuvant chemotherapy alone, adding immunotherapy resulted in a higher pCR rate, DFS/PFS/EFS/RFS, and OS without increasing toxicity rates. |
| Zhang et al.2024 (11) | To compare the efficacy and safety of neoadjuvant chemo-immunotherapy with chemotherapy alone in resectable NSCLC patients with negative PD-L1 expression. | 6 | RCTs | 3194 | EFS, OS, MPR, and pCR | Neoadjuvant chemo-immunotherapy significantly improved the pathological response and EFS in patients with PD-L1<1%. |
| Wang et al. 2024 (12) | To explore the efficacy and safety of different neoadjuvant immunotherapy regimens and cycles in NSCLC. | 44 | RCTs and cohort studies | 2430 | MPR, pCR, R0 resection rate, and AEs | Compared to neoadjuvant immunotherapy alone, neoadjuvant immunochemotherapy significantly enhanced the pathological response rate while increasing the incidence of AEs. The efficacy and safety of three cycles of neoadjuvant chemoimmunotherapy were better than two and four cycles. |
| Wang et al. 2025 (13) | To explore the efficacy and safety of different neoadjuvant immunotherapy combinations in early-stage NSCLC. | 9 | RCTs | 3431 | MPR, pCR, EFS, surgical resection rate, R0 resection rate, and AEs of grade 3 or greater | Neoadjuvant toripalimab plus chemotherapy demonstrated better survival benefits compared to other regimens， but it also increased the rate of serious AEs. |
| Ye et al. 2024 (14) | To explore the efficacy and safety of different cycles of neoadjuvant immunotherapy in resectable NSCLC. | 29 | RCTs, single-arm studies, and observational studies | 1331 | MPR, pCR, complete response, partial response, surgical delay rate or R0 resection rate, and AEs | Compared to two cycles of neoadjuvant immunotherapy, three cycles of regimens only improved the radiographic response rates but not the pathological response rates. |
| He et al. 2024 (15) | To compare the efficacy and safety of neoadjuvant immunochemotherapy with perioperative immunotherapy in operable NSCLC patients | 7 | RCTs | 2934 | EFS, pCR, and MPR | Perioperative immunotherapy and neoadjuvant immunochemotherapy demonstrate comparable efficacy in improving EFS. Patients with non-squamous cell carcinoma, PD-L1 expression ≥50%, or stage III disease benefit more from neoadjuvant immunochemotherapy. |
| Dong et al. 2024 (16) | To compare the efficacy of neoadjuvant ICIs on lung squamous cell carcinoma and adenocarcinoma. | 22 | RCTs and single-arm studies | 430 | MPR, pCR, and radiological responses | Lung squamous cell carcinoma patients showed better radiological response rates than those with adenocarcinoma, but there was no significant difference in pathological response rates. |
| Zhang et al. 2024 (17) | To explore the efficacy of neoadjuvant immunotherapy plus chemotherapy in resectable NSCLC. | 7 | RCTs | 2929 | EFS, OS, MPR, and pCR | Neoadjuvant immunotherapy combined with chemotherapy can significantly improve the pCR, MPR, EFS, and OS of resectable NSCLC compared to neoadjuvant chemotherapy. |
| Wang et al. 2022 (18) | To compare the efficacy and safety of neoadjuvant ICI with neoadjuvant chemotherapy in resectable NSCLC. | 51 | Single-arm or multi-arm trials | 3380 | MPR, pCR, ORR, grade ≥3 TRAEs, surgical resection rate, R0 resection rate, and surgical complication | Compared to neoadjuvant chemotherapy and neoadjuvant ICI alone, neoadjuvant ICI combination therapy significantly improved MPR and pCR rates. PD-1 inhibitors appear to be associated with superior efficacy compared to PD-L1 inhibitors. PD-L1 expression demonstrated predictive value for MPR and pCR in patients receiving neoadjuvant ICI. |

DFS, disease-free survival; OS, overall survival; EFS, event-free survival; pCR, pathological complete response; MPR, major pathological response; AEs, adverse events; RCTs, randomized controlled trials; TRAEs, treatment-related adverse events; ICIs, immune checkpoint inhibitors; ORR, objective response rate; RF, recurrence-free survival; PFS, progression-free survival; PD-L1, programmed cell death ligand 1; PD-1, programmed death 1; irAEs, immune-related adverse events.

**References**

1.Zhang W, Liang Z, Zhao Y, Li Y, Chen T, Li W, et al. Efficacy and safety of neoadjuvant immunotherapy plus chemotherapy followed by adjuvant immunotherapy in resectable non-small cell lung cancer: a meta-analysis of phase 3 clinical trials. Frontiers In Immunology. 2024;15:1359302.

2.Li Z, Zhang X, Wang Y, Yu Z, Yang C, Zhou Y, et al. Adjuvant therapy in completely resected, EGFR-mutant non-small cell lung cancer: a comparative analysis of treatment efficacy between EGFR-TKI and anti-PD-1/PD-L1 immunotherapy. J Immunother Cancer. 2023;11(10).

3.Zhou Y, Li A, Yu H, Wang Y, Zhang X, Qiu H, et al. Neoadjuvant-Adjuvant vs Neoadjuvant-Only PD-1 and PD-L1 Inhibitors for Patients With Resectable NSCLC: An Indirect Meta-Analysis. JAMA Netw Open. 2024;7(3):e241285.

4.Nuccio A, Viscardi G, Salomone F, Servetto A, Venanzi FM, Riva ST, et al. Systematic review and meta-analysis of immune checkpoint inhibitors as single agent or in combination with chemotherapy in early-stage non-small cell lung cancer: Impact of clinicopathological factors and indirect comparison between treatment strategies. Eur J Cancer. 2023;195:113404.

5.Han Y, Xiao X, Qin T, Yao S, Liu X, Feng Y, et al. Efficacy and safety of perioperative immunotherapy combinations for resectable non-small cell lung cancer: a systematic review and network meta-analysis. Cancer Immunol Immunother. 2024;73(12):262.

6.Chen Y, Qin J, Wu Y, Lin Q, Wang J, Zhang W, et al. Does major pathological response after neoadjuvant Immunotherapy in resectable nonsmall-cell lung cancers predict prognosis? A systematic review and meta-analysis. Int J Surg. 2023;109(9):2794-807.

7.Pasqualotto E, Moraes FCA, Chavez MP, Souza MEC, Rodrigues A, Ferreira ROM, et al. PD-1/PD-L1 Inhibitors plus Chemotherapy Versus Chemotherapy Alone for Resectable Non-Small Cell Lung Cancer: A Systematic Review and Meta-Analysis of Randomized Controlled Trials. Cancers (Basel). 2023;15(21).

8.Meng Y, Zhang Q, Wu R, Li H, Wang Z, Yao Y, et al. Efficacy and safety of perioperative, neoadjuvant, or adjuvant immunotherapy alone or in combination with chemotherapy in early-stage non-small cell lung cancer: a systematic review and meta-analysis of randomized clinical trials. Ther Adv Med Oncol. 2024;16:17588359241284929.

9.Zhang Q, Duan J, Zhang Y, Yang L, Li D. Perioperative or neo/adjuvant chemoimmunotherapy versus chemotherapy for resectable non-small cell lung cancer: a systematic review and network meta-analysis. Syst Rev. 2025;14(1):24.

10.Wu Y, Verma V, Gay CM, Chen Y, Liang F, Lin Q, et al. Neoadjuvant immunotherapy for advanced, resectable non-small cell lung cancer: A systematic review and meta-analysis. Cancer. 2023;129(13):1969-85.

11.Zhang SL, Tian Y, Yu J, Zhang JH, Sun L, Huang LT, et al. Is neoadjuvant immunotherapy necessary in patients with programmed death ligand 1 expression-negative resectable non-small cell lung cancer? A systematic review and meta-analysis. Lung Cancer. 2024;191:107799.

12.Wang H, Liang S, Yu Y, Han Y. Efficacy and safety of neoadjuvant immunotherapy protocols and cycles for non-small cell lung cancer: a systematic review and meta-analysis. Frontiers In Oncology. 2024;14:1276549.

13.Wang L, Zheng G, Hu Y, Maolan A, Luo Y, Li Y, et al. Comparative efficacy and safety of first-line neoadjuvant therapy for early-stage non-small cell lung cancer based on immune checkpoint inhibitor therapy: a systematic review and network meta-analysis. BMC Pulm Med. 2025;25(1):49.

14.Ye L, Liu Y, Xiang X, Wang Z, Peng W, Wei X, et al. Efficacy and safety of different cycles of neoadjuvant immunotherapy in resectable non-small cell lung cancer: A systematic review and meta-analysis. Heliyon. 2024;10(11):e31549.

15.He Z, Zhu Q, Xia X, Wu J, Xiao H, Qiao G, et al. Immunotherapy in operable non-small cell lung cancer: a systematic review and network meta-analysis of efficacy between neoadjuvant immunochemotherapy and perioperative immunotherapy. J Thorac Dis. 2024;16(10):6699-712.

16.Dong J, Wang Q, Wang R, Ye K, Ye Z, Lin J, et al. The efficacy of neoadjuvant immune checkpoint inhibitors in lung squamous cell carcinoma and adenocarcinoma: a systematic review and single-arm meta-analysis. J Thorac Dis. 2024;16(10):6918-35.

17.Zhang W, Dai T, Wang D, Zhu Y, Hua W. Efficacy of neoadjuvant PD-1/PD-L1 inhibitor in resectable NSCLC: a meta-analysis based on randomized controlled trials. BMC Cancer. 2024;24(1):1522.

18.Wang H, Liu T, Chen J, Dang J. Neoadjuvant immunotherapy and neoadjuvant chemotherapy in resectable non-small cell lung cancer: A systematic review and single-arm meta-analysis. Front Oncol. 2022;12:901494.
